# Patterns of molecular and phenotypic diversity in pearl millet [*Pennisetum glaucum *(L.) R. Br.] from West and Central Africa and their relation to geographical and environmental parameters

**DOI:** 10.1186/1471-2229-10-216

**Published:** 2010-10-06

**Authors:** Benjamin Stich, Bettina IG Haussmann, Raj Pasam, Sankalp Bhosale, C Tom Hash, Albrecht E Melchinger, Heiko K Parzies

**Affiliations:** 1 Max Planck Institute for Plant Breeding Research, Carl-von-Linné Weg 10, 50829 Köln, Germany; 2 International Crops Research Institute for the Semi-Arid Tropics (ICRISAT), Niamey, BP 12404, Niamey, Niger; 3 Institute of Plant Breeding, Seed Science, and Population Genetics, University of Hohenheim, 70593 Stuttgart, Germany; 4 ICRISAT, Patancheru, Hyderabad 502324, Andhra Pradesh, India

## Abstract

**Background:**

The distribution area of pearl millet in West and Central Africa (WCA) harbours a wide range of climatic and environmental conditions as well as diverse farmer preferences and pearl millet utilization habits which have the potential to lead to local adaptation and thereby to population structure. The objectives of our research were to (i) assess the geographical distribution of genetic diversity in pearl millet inbreds derived from landraces, (ii) assess the population structure of pearl millet from WCA, and (iii) identify those geographical parameters and environmental factors from the location at which landraces were sampled, as well as those phenotypic traits that may have affected or led to this population structure. Our study was based on a set of 145 inbred lines derived from 122 different pearl millet landraces from WCA.

**Results:**

Five sub-groups were detected within the entire germplasm set by STRUCTURE. We observed that the phenotypic traits flowering time, relative response to photoperiod, and panicle length were significantly associated with population structure but not the environmental factors which are expected to influence these traits in natural populations such as latitude, temperature, or precipitation.

**Conclusions:**

Our results suggested that for pearl millet natural selection is compared to artificial selection less important in shaping populations.

## Background

Pearl millet [*Pennisetum glaucum *(L.) R. Br.] is an annual, diploid, highly allogamous cereal with seven chromosome pairs [1]. It can be grown in a vast range of environmental conditions including environments that are characterized by frequent drought events and poor soil fertility [2]. This is one reason that pearl millet is one of the most important staple food crops in West and Central Africa (WCA) [3]. The other reason is that pearl millet grain has relatively high nutritional values for a cereal. Its grain has higher protein and fat content than wheat or rice and its amino acid composition is more appropriate for human nutrition than that of wheat or polished rice [4-6].

Cultivated pearl millet displays tremendous phenotypic variability for traits such as flowering time, panicle length, grain and stover characteristics, tolerance to drought, pests, and diseases, as well as nutritional value (e.g., [7]). Efficient and systematic exploitation of this diversity is the key to any crop improvement program [8]. This, however, requires in a first step the assessment of genetic diversity and population structure of the species under consideration.

For pearl millet, several studies have examined these issues. [9] determined the influence of farmer management on pearl millet landrace diversity in two villages in North-Eastern Nigeria. [10] assessed the genetic diversity within and between ten Indian pearl millet landraces. The genetic diversity of 46 wild and 421 cultivated genotypes of pearl millet from Niger was analyzed by [11,12] examined the phylogeny and origin of pearl millet. However, to our knowledge, no earlier study examined the genetic diversity and population structure of pearl millet across a wide geographic range in WCA.

The distribution area of pearl millet in WCA harbours a wide range of climatic and environmental conditions as well as diverse farmer preferences and pearl millet utilization habits (cf. [13]). This may lead to local adaptation and thereby to population structure. However, no earlier study examined systematically the forces that may have affected or led to the observed population structure in pearl millet.

The objectives of our research were to (i) assess the geographical distribution of genetic diversity, (ii) assess the population structure of pearl millet from WCA, and (iii) identify those geographical parameters and environmental factors from the location at which landraces were sampled, as well as those phenotypic traits that may have affected or led to this population structure.

## Results

**Figure 1:**
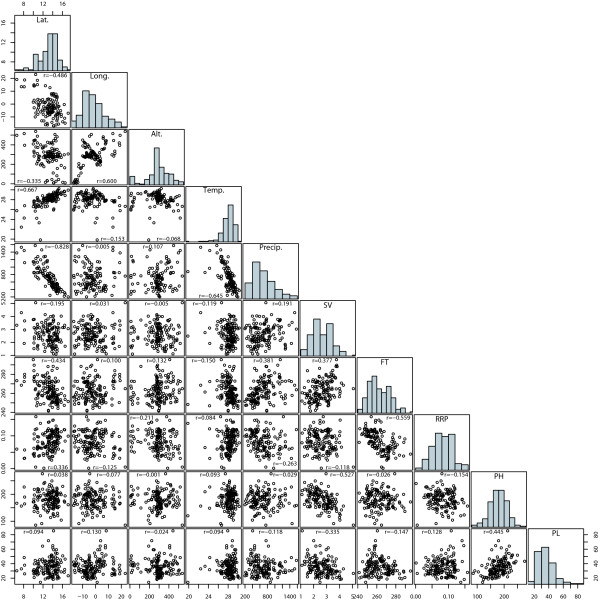
**Distributions and correlations between the geographic and environmental parameters, as well as the evaluated phenotypic traits for the 145 pearl millet genotypes**. For a detailed definition of the examined indicators see Materials and Methods.

The heritabilities of the four phenotypic traits assessed for the 145 pearl millet in-breds ranged from 0.64 for SV to 0.93 for PL and was 0.80 for PH and 0.89 for FT. The geographic and environmental parameters as well as the phenotypic traits available for all pearl millet inbreds showed a continous distribution (Figure 1). The correlation between the geographic and environmental parameters and examined phenotypic traits ranged from -0.828 between latitude and precipitation to 0.667 between latitude and mean annual temperature.

For the 20 SSR markers examined in our study, the number of alleles ranged from 9 to 28 (Table 1), with an average of 16.4. Gene diversity D was lowest for markers PSMP2249 and PSMP2267 (0.49) and highest for marker PSMP2063 (0.92). The average D value across all pearl millet inbreds was 0.74.

**Table 1:** Genetic characteristics of the simple sequence repeat markers used to analyze the pearl millet inbreds, where N is the number of successfully genotyped pearl millet inbreds and D the gene diversity.

Marker	N	No. alleles	**Major allele freq**.	D
PSMP2001	122	17	0.38	0.78
PSMP2008	137	21	0.22	0.89
PSMP2027	129	22	0.25	0.88
PSMP2030	131	12	0.32	0.78
PSMP2043	125	12	0.49	0.71
PSMP2063	128	28	0.15	0.92
PSMP2071	112	14	0.22	0.87
PSMP2076	119	17	0.46	0.74
PSMP2080	111	21	0.19	0.90
PSMP2085	131	14	0.38	0.76
PSMP2087	124	11	0.48	0.69
PSMP2090	130	20	0.34	0.83
PSMP2208	128	22	0.60	0.62
PSMP2237	137	20	0.35	0.79
PSMP2246	116	23	0.48	0.70
PSMP2248	127	9	0.48	0.68
PSMP2249	132	10	0.69	0.49
PSMP2267	127	15	0.71	0.49
PSMP2275	136	10	0.46	0.66
ICMP3002	137	10	0.59	0.53

**Figure 2:**
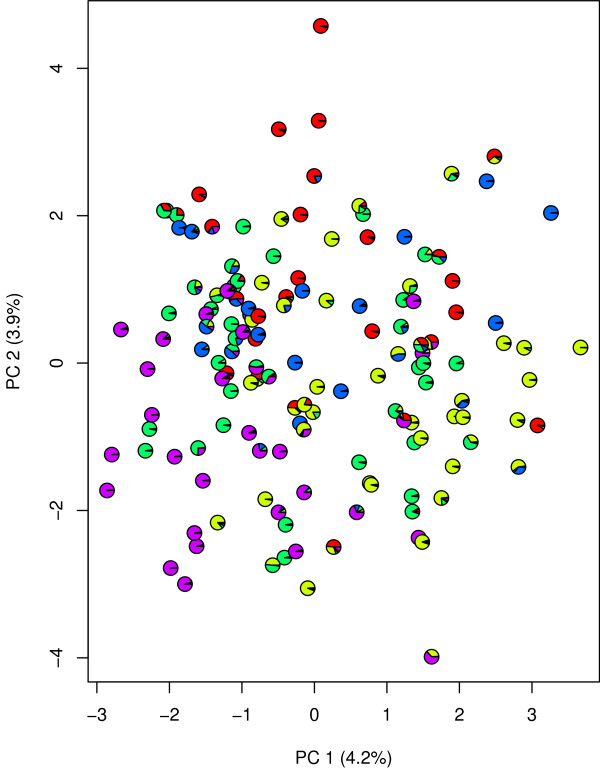
**Principal component analysis of the 145 pearl millet inbreds examined in our study based on the simple sequence repeat marker allele frequencies**. PC1 and PC2 are the first and second principal component, respectively, and the values in brackets give the proportion of explained variance. The different colored segments of the pie charts give the probability that a certain individual belongs to one of the five sub-groups identified by STRUCTURE.

The first two principal components, which explained 4.2 and 3.9% of the total genetic variation, revealed no obvious clusters (Figure 2). The STRUCTURE analysis resulted in five sub-groups and one admixed group. These groups, which comprised between 14 and 40 inbreds (Table 2), were located in different sectors of the PCA. The gene diversity D of the sub-groups ranged from 0.62 (sub-group 1) to 0.72 (sub-group 4) and the number of group-specific alleles varied between 15 (sub-group 3) and 44 (sub-group 4). The overall fixation index F*_st _*was 0.08. The number of alleles per locus of the pearl millet subsets of size 5, 10, 15, ..., 40 which maximize the gene diversity D ranged from 4.5 to 12.5, where gene diversity D varied between 0.74 and 0.82 (Table 3).

**Table 2:** Genetic diversity of the pearl millet inbreds with respect to their country of origin, their agro-ecological zone of origin, and their sub-groups identified by STRUCTURE, where N is the population size, D the gene diversity, and A the number of group-specific alleles.

Hierarchy level	Name	N	Alleles/locus	D	A
Country	Benin	5	3.4	0.62	4
	Burkina Faso	27	8.0	0.70	32
	Cameroon	9	4.7	0.67	9
	Central African Republic	4	3.0	0.57	6
	Chad	1	1.0	-	0
	Guinea	3	2.3	0.51	3
	Mali	50	10.4	0.72	45
	Mauritania	4	3.1	0.60	9
	Niger	29	8.1	0.71	26
	Senegal	13	5.6	0.69	9
					
Agro-ecological zone	Sahelian zone	43	9.3	0.72	38
	Sudano-sahelian zone	74	12.2	0.72	82
	Sudanian zone	28	8.8	0.73	37
					
STRUCTURE	Sub-group 1	16	5.1	0.62	16
	Sub-group 2	25	6.1	0.65	23
	Sub-group 3	25	5.7	0.65	15
	Sub-group 4	14	6.4	0.72	44
	Sub-group 5	25	6.3	0.68	22
	Admixed group	40	8.9	0.71	32

	Total	145	16.4	0.74	

**Table 3:** Properties of the pearl millet subsets maximizing gene diversity D.

	Subset size							
	
	5	10	15	20	25	30	35	40
D	0.74	0.81	0.82	0.82	0.82	0.82	0.82	0.81
Alleles/locus	4.5	7.4	8.5	9.8	10.6	11.2	12.2	12.5
Composition								
Country								
Benin	0	0	1	1	2	2	2	2
Burkina Faso	1	2	1	2	1	4	5	7
Cameroon	0	1	0	2	3	3	2	3
Central African Republic	0	0	0	0	0	1	0	0
Chad	0	0	0	0	0	0	0	0
Guinea	0	0	1	2	2	2	2	2
Mali	2	4	6	5	6	8	10	13
Mauritania	0	1	2	3	3	3	3	3
Niger	2	2	4	5	6	4	9	7
Senegal	0	0	0	0	2	3	2	3
Agro-ecological zone								
Sahelian zone	2	4	6	6	9	7	12	9
Sudano-sahelian zone	3	5	5	10	11	12	14	19
Sudanian zone	0	1	4	4	5	11	9	12

**Figure 3:**
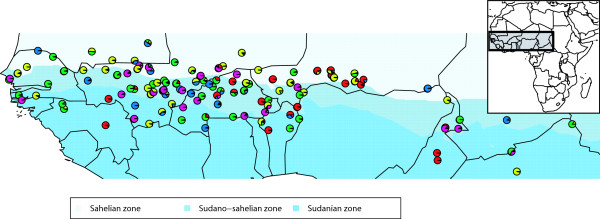
**Geographical projection of the membership probability that each of the 145 pearl millet inbreds belongs to one of the five sub-groups identified by STRUCTURE**.

The AMOVA with the country of origin as hierarchy level revealed that most of the genetic variation was present between inbreds derived from landraces of the same country as well as within landraces but only a small proportion of the total genetic variation was attributable to countries (Additional file 1). The same trend was observed with respect to the agro-ecological zone of origin as hierarchy level (Additional file 1). Plotting the STRUCTURE results on the geographic map revealed no obvious association of sub-group membership probability and country of origin or agro-ecological zone of origin (Figure 3).

The pairwise Pearson's correlation coefficient between MRD and the geographic or phenotypic trait-based distances was highest for SBD (0.220) and lowest for PH (-0.028) (Table 4). The highest correlation with SBD was observed for PL (0.077) and the lowest for annual temperature (-0.023). The tests of association between the Q matrix from STRUCTURE and geographic or environmental parameters as well as phenotypic traits were significant (*α *= 0.05) for FT, RRP, PL, and country of origin.

**Table 4:** Pairwise Pearson's correlation coefficient *ρ *between the modified Rogers distance (MRD) or the STRUCTURE based distance (SBD) of the 145 pearl millet genotypes and the corresponding geographic distance, the environmental distance calculated from geographical or environmental parameters as well as the evaluated phenotypic traits.

	*ρ*		P value^†^
		
	MRD	SBD	
SBD	0.220***		
Geographic dist.	0.070***	0.037*	
Latitude	0.015	0.006	0.1355
Longitude	0.070***	0.037*	0.1682
Altitude	0.089***	0.038*	0.0683
Temperature	-0.023*	-0.023*	0.1668
Precipitation	0.040**	0.050**	0.2001
Seedling vigor	0.017	0.051**	0.2623
Flowering time	0.045**	0.070***	0.0035
Relative response to photoperiod	0.031*	0.035*	0.0029
Plant height	-0.028*	-0.011	0.1584
Panicle length	0.019*	0.077***	0.0010
Country			0.0056
Agro-ecological zone			0.1139

* P < 0.05, ** P < 5 × 10^−5^, *** P < 5 × 10^−10^

^†^P value for the association of the corresponding parameters with the Q matrix from STRUCTURE based on a multivariate linear regression.

## Discussion

### Genetic diversity of the examined pearl millet germplasm

Irrespective of the considered hierarchy level, the AMOVA revealed that about four times more variation was present between landraces than within landraces (Additional file 1). Our finding is in good accordance with the results of [10] who observed that about 2.5 times more variation was present between Indian pearl millet landraces than within the landraces. Consequently, we observed realistic estimates of within and between landrace variation despite the fact that we examined inbreds derived from landraces as well as that the average number of inbreds per landrace was only 1.2.

Long-term selection gain requires genetic variability [8]. Therefore, it was important to examine the genetic diversity of the pearl millet inbreds analyzed in our study. Since estimates of genetic diversity D are not affected by differences in sample size, direct comparisons between different studies are possible. Across the 145 pearl millet inbreds examined, we observed a total gene diversity D of 0.74 (Table 2). This value is higher than the gene diversity estimates observed in the study of [11] (0.49) for 421 genotypes of 140 allogamous cultivated pear millet landracs from Niger, based on SSR marker. This difference might be explained by the fact that in the latter study only genotypes from Niger were examined, whereas the inbreds of our study were derived from landraces from a much larger area of WCA. Furthermore, we observed a higher D value than [12] (0.60) for a pearl millet world collection. This finding might be due to the fact that we used a higher proportion of di-nucleotide repeat SSR markers, which tend to be more variable than SSRs with longer repeat motifs (cf., [14]), than [12].

Our observation on D was supported by the results on the average number of alleles per locus. Although our population size was smaller than that of the other studies and we examined inbreds instead of heterozygous genotypes, we observed a considerably higher number of alleles per locus (16.4) (Table 2) than previously re-ported by [11] (6.2) and [12] (9.6). These findings on D and the average number of alleles per locus suggested that the pearl millet inbreds examined in this study is a valuable resource for increasing the genetic diversity in pearl millet breeding pro-grams.

### Spatial distribution of genetic diversity

We observed differences between the genetic diversity parameters gene diversity D, average number of alleles per locus, and the number of group specific alleles A between the pearl millet inbreds originating from different WCA countries (Table 2). However, these differences are to a large extent (data not shown) only due to the fact that the number of inbreds originating from each of the ten countries differed considerably. Furthermore, the AMOVA revealed that less than one percent of the variation was found between countries (Additional file 1). This observation is in contrast to findings of [15], who observed statistically different D values for pearl millet genotypes from different countries. In addition, that study reported coefficients of differentiation between the genotypes from different countries (0.07-0.23) that suggested the presence of considerable variation between countries. These contrasting findings compared to our results might be due to the fact that our sampling was extremely unbalanced with respect to the number of inbreds per country. Another explanation might be that our study was based on SSR markers, whereas [15] examined isozyme markers that may not be selectively neutral and reveal only a low number of alleles per locus.

In WCA, pearl millet is cultivated throughout three agro-ecological zones [16]. The adaptation of pearl millet to these different environments has the potential to lead to genetic differentiation. However, AMOVA revealed that almost none of the SSR genotype variation observed could be attributed to differences between the agro-ecological zones (Additional file 1). Our observation suggested that establishing core-collections based on the information of the agro-ecological zone of origin (e.g., [7]) might be sub-optimal. This was supported by our observation that the three agro-ecological zones contributed different numbers of inbreds to the pearl millet subsets maximizing the gene diversity D (Table 3).

Our findings might be explained by the habit of farmers, especially in the sudano-sahelian and in the sudanian zones, to grow not only one landrace but several (Bettina I.G. Haussmann, personal communication). In order to fill the hungry period, an early maturing landrace is cultivated, and in addition a landrace with late maturity that has a high yield potential in years with good growing conditions but might fail in years with severe terminal drought. Also the highly allogamous behaviour of the crop combined with an overlapping flowering time of early and late but photo-sensitive landraces in certain years with late planting dates has the potential to diluting differentiation between agro-ecological zones. Finally, our observation might be explained by the fact that the environmental conditions within each agro-ecological zone are too heterogeneous (high inter-annual climate variability) to permit detectable genetic differentiation between landraces of different agro-ecological zones.

### Inference of population structure

Due to the fact that neither country nor agro-ecological zone of origin revealed a clear sub-grouping of the pearl millet inbreds examined in our study (Additional file 1). we used the software STRUCTURE [17] to infer the population structure. The results of this analysis indicated that the 145 pearl millet inbreds of our study belong to five sub-groups (Additional file 2). The grouping of the pearl millet inbreds by STRUCTURE was in fair accordance with the results of the PCA (Figure 2). the five groups showed no distinct clusters but were located in different sectors of the PCA. Our finding together with an overall F*_st _*value of 0.08 suggested that the sub-groups of pearl millet are not as differentiated as in maize (e.g., [18]).

Our finding of five sub-groups for pearl millet inbreds from WCA was in the range of previously reported numbers of sub-groups. [15] identified based on isozyme markers two distinct sub-groups in pearl millet inbreds from West Africa, whereas [19] reported three sub-groups for pearl millet landraces from Niger. In contrast, [1] identified based on phenotypic data of morphological and disease resistance traits ten clusters of pearl millet landraces from Burkina Faso. These differences in the number of sub-groups compared to our study, are most likely due to the fact that earlier studies examined a lower number of accessions than we did. In addition, the above mentioned studies were based either on phenotypic data or a relatively low number of isozyme markers, while we used SSR markers to examine pearl millet inbreds.

### Association of population structure and geographical, environmental, and phenotypic parameters

In the harshest production environments in WCA, farmers rely in highly cross-pollinated pearl millet landraces with site-specific adaptation and good production stability that are rarely outyielded by on-station-bred improved cultivars [20]. Thereby, farmers, together with the environmental factors, shape the pearl millet populations (cf. [9,21]). Identifying the variables that are correlated with population structure, thus, has the potential to help identify adaptive traits as well as the environmental conditions which are the driving factors of adaptation.

We observed that the phenotypic traits FT, PPR, and PL were significantly associated with the Q matrix from STRUCTURE (Table 4) using a multivariate linear regression model. For FT, our observation is in accordance with observations in other plant species such as maize [22,23] or Arabidopsis [24]. This finding can be explained by the fact that FT is in most plant species under divergent selection as it is the key adaptive trait enabling plants to flower at the optimum time for pollination and seed development [25].

In WCA, the growing conditions of pearl millet are characterized, among other hazards, by highly variable beginnings of the rainy season [26]. Photoperiod-sensitive flowering, in our study measured as PPR, has the potential to enhance adaptation to such environments. This is due to the fact that it leads to simultaneous flowering of genotypes in the target region, independent of the individual date of sowing in different fields. This in turn, is expected to lead to divergence and thereby might explain why PPR is associated with population stucture in pearl millet (Table 4).

Our finding that PL was significantly associated with the Q matrix from STRUCTURE (Table 4) might be explained by the fact that farmers preferences seem to differ largely in different regions of WCA (cf. [27]). This has the potential to lead to population stucture. The preference for a long panicle in certain regions of WCA is mostly due to practical reasons. In these areas, the harvested pearl millet panicles are usually tied up into bundles for transport from the field to the grain store, and this can be more easily done with long panicles, where short panicles require usually a bag for transportation.

We observed that the phenotypic traits FT, RRP, and PL were significantly associated with the Q matrix from STRUCTURE, but not the environmental factors (Table 4) that are expected to influence these traits in natural populations such as latitude, temperature, or precipitation (also not the monthly averages; data not shown). This was also true, if the phenotypic traits were used as cofactors when examining the environmental factors (data not shown). Our results suggested that for pearl millet landraces natural selection is less important than human selection in shaping populations which in turn can be explained by the fact that pearl millet landraces are no natural populations.

## Conclusions

Our findings of high D values as well as a high average number of alleles per locus suggested that the pearl millet inbreds examined in this study is a valuable resource for increasing the genetic diversity in pearl millet breeding programs. Furthermore, the results of this study suggested that for pearl millet landraces natural selection is less important than human selection in shaping populations.

## Methods

### Plant materials

A set of 145 inbred lines (14 inbreds in S3, 131 inbreds in S4), derived from 122 different pearl millet landraces, were used in this study (Additional file 3). Our study was based on inbred lines derived from landraces as for this type of germplasm phenotypic evaluation can be performed on the basis of several plants per genotype and, thus, with higher heritability. Landraces were considered as different, if they have a different local name or were collected from different locations (cf. [9]). The number of inbreds per landrace ranged from one to two and was on average 1.2. The landraces had been assembled during joint pearl millet collection missions involving the "Institut de la Recherche pour le Développement" (IRD) and International Crops Research Institute for the Semi-Arid Tropics (ICRISAT) in 1976 and 2003, and were obtained by ICRISAT Niger in 2005 from IRD Montpellier. The geographic origin of the landraces covered 117 villages located in the following ten WCA countries: Benin, Burkina Faso, Cameroon, Central African Republic, Chad, Guinea, Mali, Mauritania, Niger, and Senegal. From each collection site, the geographic coordinates were recorded using a GPS device.

### Environmental parameters and phenotypic evaluation

For each collection site, mean annual precipitation and mean annual temperature were calculated using the gridded bivariate interpolation method [28] based on more than 160 years of data available from ftp://ftp.ncdc.noaa.gov/pub/data/ghcn/v2/. Based on the mean annual precipitation, each collection site was assigned to one of three agro-ecological zones (sahelian, sudano-sahelian, sudanian) [16]. In addition, the altitude of each collection site was obtained by cross-referencing the geographic coordinates with the WORLDCLIM database [29] using DIVA-GIS software [30].

All 145 pearl millet inbreds were grown in 2007 on the ICRISAT research station in Sadore (Niger) with two sowing dates (15*^th ^*June and 16*^th ^*July). The two experiments were located next to each other. The design of each experiment was an *α*-lattice with two replicates. As experimental units, one-row plots with a length of 4.8 m and with 0.75 m between rows, were used. The recorded traits were seedling vigor (SV, score from 1 to 5, 1 = best, 5 = worst), flowering time (FT, Julian days), plant height (PH, cm), and panicle length (PL, cm). For each trait, the adjusted entry mean across both sowing dates, where heterogeneous error variances were assumed, was calculated. Heritability *h*^2 ^was computed as:

$$h^{2}=\frac{\sigma^{2}{}_{g}}{\sigma^{2}{}_{g}+\bar{v}/2},$$

where *σ*^2^*_g _*is the genotypic variance and $\bar{v}/2$ the mean variance of a difference of two adjusted treatment means [31]. Furthermore, for each inbred, the relative response to photoperiod (RRP) was calculated as 1-(FT_2_/FT_1_) [32], where FT_1 _and FT_2 _were the adjusted entry means for FT (Julian days) observed in the first and second experiment, respectively. This parameter is unit-less and deviates from 0 for genotypes responding to photoperiod.

### Molecular markers

Total genomic DNA was extracted from leaf tissue using a modified CTAB protocol [33]. A total of 20 simple sequence repeat markers (SSRs) [34-37] (Additional file 4) were used to genotype the 145 pearl millet inbreds. The SSRs were grouped into multiplex sets of three, where forward primers were labeled with fluorescent dyes (6-FAM, HEX, and TET; Biomers GmbH, Germany), and amplicons were generated using an amplification program of 94°C/3 min, followed by 30 cycles of 94°C/45 s, optimum annealing temperature T*_opt_*/1 min (Additional file 4) and 72°C/45 s, and a final extension step of 72°C/10 min. PCR products were denatured and size-fractioned using capillary electrophoresis on a MegaBACE sequencer (Amersham Biosciences, Sweden). The MegaBACE Fragment Profiler v1.2 (Amersham Biosciences, Sweden) was applied to size peak patterns, using internal ROX 400 HD for allele calling (Additional file 5). Each of the 20 SSRs showed less than 25% missing values. The map positions of these markers were extracted from the Gramene database.

### Statistical analyses

Due to the genome-wide distribution of the SSR markers used in this study (Additional file 4) as well as the rapid decay of linkage disequilibrium in pearl millet [38], linkage disequilibrium between markers was neglected for all statistical analyses described below. The number of alleles per locus, the number of group-specific alleles A, and the gene diversity D [39] were determined. Modified Rogers distance (MRD) was calculated according to [40] and an F*_st _*analysis was performed according to [39] using the observed and expected heterozygosities for the population under consideration. Principal component analysis (PCA) of the 145 inbreds based on the (i) SSR allele frequency matrix and (ii) geographical and environmental parameters as well as the evaluated phenotypic traits (Additional file 6) was carried out. Analyses of molecular variance (AMOVA) were performed using Arlequin [41].

In order to identify those *r *= 5, 10, 15, ..., 40 pearl millet inbreds which maximize the gene diversity D, we used an algorithm which is based on an iterative maximization procedure [42]. Briefly, a subset of *r *inbreds was first chosen at random from the entire 145 pearl millet inbreds. In step one, all the subsets of size (*r *- 1) were examined. The subset having the highest level of D was retained. In step two, among the remnant inbreds, the inbred bringing the greatest increase in D was added. These two steps were repeated until the gene diversity D of the subset reached a maximum.

A model-based approach implemented in software package STRUCTURE [17] was used to determine the presence of population structure and assign pearl millet inbred lines to sub-groups. In our investigations, the set of 145 inbreds was analyzed by setting the number of sub-groups from one to 20 with five repetitions. For each run of STRUCTURE, the burn-in time as well as the iteration number for the Markov chain Monte Carlo algorithm was set to 100,000. We used the *ad hoc *criterion described by [43] to estimate the number of sub-groups. From the five repetitions with the estimated number of sub-groups, the one with the maximum likelihood was used to assign lines with membership probabilities of 0.80 or more to sub-groups. Inbreds with membership probabilities less than 0.80 for all individual sub-groups were assigned to an admixed group.

Pairwise geographic distances between all 145 pearl millet inbreds were calculated from the geographic coordinates. Furthermore, for each geographic and environmental parameter, phenotypic trait, as well as for the Q matrix from STRUCTURE (SBD), distances *ED *between all pairs of pearl millet inbreds were calculated as:

$$ED_{ij}={\sqrt{\sum_{k=1}^{n}\left(\frac{t_{ik}-t_{k}^{-}}{\sigma t_{k}}-\frac{t_{jk}-t_{k}^{-}}{\sigma t_{k}}\right)}}^{2},$$

where *ED_ij _*is the distance between inbred *i *and *j*, *n *the number of dimensions of the examined parameter, *t_ik _*and *t_jk _*the parameter values of the inbreds *i *and *j *for the *k*th dimension, $t_{k}^{-}$ the mean parameter value of the *k*th dimension across all inbreds, and $\sigma_{t_{k}}$ the standard deviation of the parameter values for the *k*th dimension across all inbreds. For the Q matrix from STRUCTURE, *n *= 4, where *n *= 1 for the other parameters.

Pearson's correlation coefficient was calculated for all combinations between MRD and the above mentioned distances as well as between SBD and the above mentioned distances. In addition, we used the following multivariate linear regression model:

$$Q_{il}=\mu_{l}+t_{i}+e_{il,}$$

where *Q_il _*is the probability that the *i*th pearl millet inbred belongs to the *l*th sub-group (*i.e*., the value of the *i*th row in the *l*th column of the Q matrix from STRUCTURE), *μ_l _*the intercept term for the *l*th column of the Q matrix from STRUCTURE, *t_i _*the parameter value of the *i*th pearl millet inbred, and *e_il _*the residual. We examined the geographic or environmental parameters as well as phenotypic traits with this model in order to identify those parameterrs which explain best the variation in the Q matrix from STRUCTURE.

If not stated differently, all analyses were performed with the statistical software R [44].

## Authors' contributions

BS, BIGH, and HKP designed the study. RP, SB, and BIGH carried out the molecular marker and phenotypic evaluation work. BS performed the analyses. BS, BIGH, CTH, AEM, and HKP wrote the manuscript. All authors read and approved the final version of the manuscript.

## Supplementary Material

Additional file 1 **Analysis of molecular variance for the 145 pearl millet inbred genotypes of this study**. Analysis of molecular variance for the 145 pearl millet inbred genotypes with respect to their country and agro-ecological zone of origin, where DF are the degrees of freedom, SSD the sum of squares deviations, *σ*^2 ^the variance component, and % the percentage of variance contributed by each source of variation. Click here for file

Additional file 2 **Graphical representation of the results of STRUCTURE**. Graphical representation of the results of STRUCTURE, where K is the number of sub-groups. Click here for file

Additional file 3 **Details for the 145 pearl millet inbred genotypes examined in this study**. Details of the 145 pearl millet inbred genotypes examined in this study. Click here for file

Additional file 4 **Simple sequence repeats markers used in this study**. Simple sequence repeats markers used in this study, where LG is the linkage group, Pos. the position in cM, and T*_opt _*the optimized annealing temperature. Click here for file

Additional file 5 **Screen shot of the MegaBACE Fragment Profiler**. Screen shot of the MegaBACE Fragment Profiler to illustrate the procedure of allele calling. Click here for file

Additional file 6 **Principal component analysis of the 145 pearl millet inbreds examined in our study based on the corresponding geographical and environmental parameters as well as the evaluated phenotypic traits**. Principal component analysis of the 145 pearl millet inbreds examined in our study based on the corresponding geographical and environmental parameters as well as the evaluated phenotypic traits. PC1 and PC2 are the first and second principal component, respectively, and the values in brackets give the proportion of explained variance. The different colored segments of the pie charts give the probability that a certain individual belongs to one of the five sub-groups identified by STRUCTURE. Click here for file

## References

[B1] Wilson JP Burton GW Zongo JD Dicko IO Diversity among pearl-millet landraces collected in central Burkina Faso Crop Science 1990 30 40 43 10.2135/cropsci1990.0011183X003000010009x

[B2] Rachie KO Majumdar JV Pearl millet 1980 Pennsilvania State University

[B3] Poncet V Lamy F Enjalbert J Joly H Sarr A Robert T Genetic analysis of the domestication syndrome in pearl millet (*Pennisetum glaucum *L., Poaceae): inheritance of the major characters Heredity 1998 81 648 658 10.1046/j.1365-2540.1998.00445.x

[B4] Goswami AK Sharma KP Sehgal KI Nutritive value of proteins of pearl millet of high-yielding varieties and hybrids British Journal of Nutrition 1969 23 913 916 10.1079/BJN19690102 5357056

[B5] Sawaya WN Khalil JK Safi WJ Nutritional quality of pearl-millet flour and bread Plant Foods for Human Nutrition 1984 34 117 125 10.1007/BF01094839

[B6] Khairwal IS Rai KN Andrews DJ Harinarayana G Pearl millet breeding 1999 Oxford & IBH, New Dehli, India

[B7] Bhattacharjee R Khairwal IS Bramel PJ Reddy KN Establishment of a pearl millet [*Pennisetum glaucum *(L.) R. Br.] core collection based on geographical distribution and quantitative traits Euphytica 2007 155 35 45 10.1007/s10681-006-9298-x

[B8] Allard RW Principles of plant breeding 1960 John Wiley and Sons, Inc., New York

[B9] Busso CS Devos KM Ross G Mortimore M Adams WM Ambrose MJ Alldrick S Gale MD Genetic diversity within and among landraces of pearl millet (*Pennisetum glaucum *) under farmer management in West Africa Genetic Resources and Crop Evolution 2000 47 561 568 10.1023/A:1008767220320

[B10] Bhattacharjee R Bramel P Hash CT Kolesnikova-Allen MA Khairwal IS As-sessment of genetic diversity within and between pearl millet landraces Theoretical and Applied Genetics 2002 105 666 673 10.1007/s00122-002-0917-1 12582479

[B11] Mariac C Luong V Kapran I Mamadou A Sagnard F Deu M Chantereau J Gerard B Ndjeunga J Bezancon G Pham JL Vigouroux Y Diversity of wild and cultivated pearl millet accessions (*Pennisetum glaucum *[L.] R. Br.) in Niger assessed by microsatellite markers Theoretical and Applied Genetics 2006 114 49 58 10.1007/s00122-006-0409-9 17047913

[B12] Oumar I Mariac C Pham JL Vigouroux Y Phylogeny and origin of pearl millet (*Pennisetum glaucum *[L.] R. Br.) as revealed by microsatellite loci Theoretical and Applied Genetics 2008 117 489 497 10.1007/s00122-008-0793-4 18504539

[B13] Leblanc JM Pernes J Enzyme polymorphism of *Pennisetum americanum *in the Ivory-coast Japanese Journal of Genetics 1983 58 121 131 10.1266/jjg.58.121

[B14] Heckenberger M Bohn M Ziegle JS Joe LK Hauser JD Hutton M Melchinger AE Variation of DNA fingerprints among accessions within maize inbred lines and implications for identification of essentially derived varieties. I. Genetic and technical sources of variation in SSR data Molecular Breeding 2002 10 181 191 10.1023/A:1020539330957

[B15] Tostain S Riandey M Marchais L Enzyme diversity in pearl-millet (*Pennisetum glaucum*).1. West-Africa Theoretical and Applied Genetics 1987 74 188 193 10.1007/BF00289967 24241563

[B16] Saidou AA Mariac C Luong V Pham JL Bezancon G Vigouroux Y Association studies identify natural variation at PHYC linked to flowering time and morphological variation in pearl millet Genetics 2009 182 899 910 10.1534/genetics.109.102756 19433627 PMC2710168

[B17] Pritchard JK Stephens M Donnelly P Inference of population structure using multilocus genotype data Genetics 2000 155 945 959 10835412 10.1093/genetics/155.2.945 PMC1461096

[B18] van Inghelandt D Melchinger AE Lebreton C Stich B Population structure and genetic diversity in a commercial maize breeding program assessed with SSR and SNP markers Theoretical and Applied Genetics 2010 120 1289 1299 10.1007/s00122-009-1256-2 20063144 PMC2854351

[B19] Tostain S Isozymic classification of pearl-millet (*Pennisetum glaucum*, poaceae) landraces from Niger (West-Africa) Plant Systematics and Evolution 1994 193 81 93 10.1007/BF00983542

[B20] Weltzien-Rattunde E Whitaker ML Rattunde HFW Dhamotharan M Anders MM Seeds of choice. Making the most of new varieties for small farmers Oxford & IBH, New Dehli, India 1998 chap. Participatory approaches in pearl millet breeding

[B21] vom Brocke KV Christinck A Weltzien R Presterl T Geiger HH Farmers' seed systems and management practices determine pearl millet genetic diversity patterns in semiarid regions of India Crop Science 2003 43 1680 1689 10.2135/cropsci2003.1680

[B22] Thornsberry JM Goodman MM Doebley J Kresovich S Nielsen D Buckler ES Dwarf8 polymorphisms associate with variation in flowering time Nature Genetics 2001 28 286 289 10.1038/90135 11431702

[B23] Camus-Kulandaivelu L Veyrieras JB Madur D Combes V Fourmann M Barraud S Dubreuil P Gouesnard B Manicacci D Charcosset A Maize adaptation to temperate climate: relationship between population structure and polymorphism in the *Dwarf*8 gene Genetics 2006 172 2449 2463 10.1534/genetics.105.048603 16415370 PMC1456379

[B24] Zhao K Aranzana MJ Kim S Lister C Shindo C Tang C Toomajian C Zheng H Dean C Marjoram P Nordborg M An Arabidopsis example of association mapping in structured samples PLoS Genetics 2007 3 71 82 10.1371/journal.pgen.0030004 PMC1779303 17238287

[B25] Cockram J Jones H Leigh FJ O'Sullivan D Powell W Laurie DA Greenland AJ Control of flowering time in temperate cereals: genes, domestication, and sustainable productivity Journal of Experimental Botany 2007 58 1231 1244 10.1093/jxb/erm042 17420173

[B26] Haussmann BIG Boureima SS Kassari IA Moumouni KH Boubacar A Mechanisms of adaptation to climate variability in West African pearl millet landraces a preliminary assessment Journal of SAT Agricultural Research 2007 3

[B27] Haussmann BIG Boubacar A Boureima SS Vigouroux Y Multiplication and preliminary characterization of West and Central African pearl millet landraces Journal of SAT Agricultural Research 2006 2

[B28] Akima H Algorithm 761: Scattered-data surface fitting that has the accuracy of a cubic polynomial ACM Transactions on Mathematical Software 1996 22 362 371 10.1145/232826.232856

[B29] Hijmans RJ Cameron SE Parra JL Jones PG Jarvis A Very high resolution interpolated climate surfaces for global land areas International Journal of Climatology 2005 25 1965 1978 10.1002/joc.1276

[B30] Hijmans RJ Guarino L Bussink C Mathur P Cruz M Barrentes I Roja E DIVA-GIS. Vsn. 5.0. A geographic information system for the analysis of species distribution data 2004

[B31] Holland JB Nyquist WE Cervantes-Martinez CT Estimating and interpreting heritability for plant breeding: An update Plant Breeding Reviews 2003 22 9 112

[B32] White JW Laing DR Photoperiod response of flowering in diverse genotypes of common bean (*Phaseolus vulgaris*) Field Crops Research 1989 22 113 128 10.1016/0378-4290(89)90062-2

[B33] Saghai-Maroof MA Soliman KM Jorgensen RA Allard RW Ribosomal DNA spacer-length polymorphisms in barley - mendelian inheritance, chromosomal location, and population-dynamics Proceedings of the National Academy of Sciences of the United States of America 1984 81 8014 8018 10.1073/pnas.81.24.8014 6096873 PMC392284

[B34] Allouis S Qi X Lindup S Gale MD Devos KM Construction of a BAC library of pearl millet, *Pennisetum glaucum* Theoretical and Applied Genetics 2001 102 1200 1205 10.1007/s001220100559

[B35] Qi X Lindup S Pittaway TS Allouis S Gale MD Devos KM Development of simple sequence repeat markers from bacterial artificial chromosomes without subcloning BioTechniques 2001 31 355 362 11515373 10.2144/01312st08

[B36] Qi X Pittaway TS Lindup S Liu H Waterman E Padi FK Hash CT Zhu J Gale MD Devos KM An integrated genetic map and a new set of simple sequence repeat markers for pearl millet, *Pennisetum glaucum* Theoretical and Applied Genetics 2004 109 1485 1493 10.1007/s00122-004-1765-y 15322756

[B37] Senthilvel S Jayashree B Mahalakshmi V Kumar PS Nakka S Nepolean T Hash CT Development and mapping of Simple Sequence Repeat markers for pearl millet from data mining of Expressed Sequence Tags BMC Plant Biology 2008 8 119 10.1186/1471-2229-8-119 19038016 PMC2632669

[B38] Li Y Bhosale S Haussmann BIG Stich B Melchinger AE Parzies HK Genetic diversity and linkage disequilibrium of two homologous genes to maize *D8 *: Sorghum *SbD8 *and pearl millet *PgD8* Journal of Plant Breeding and Crop Science 2010 in press

[B39] Nei M Molecular evolutionary genetics 1987 Colombia University Press, New York

[B40] Wright S Evolution and genetics of populations 1978 IV The University of Chicago Press, Chicago

[B41] Excoffier L Laval G Schneider S Arlequin (version 3.0): An integrated software package for population genetics data analysis Evolutionary Bioinformatics 2005 1 47 50 PMC2658868 19325852

[B42] Gouesnard B Bataillon T Decoux G Rozale C Schoen D David J MSTRAT: An algorithm for building germ plasm core collections by maximizing allelic or phenotypic richness Journal of Heredity 2001 92 93 94 10.1093/jhered/92.1.93 11336240

[B43] Evanno G Regnaut S Goudet J Detecting the number of clusters of individuals using the software STRUCTURE: a simulation study Molecular Ecology 2005 14 2611 2620 10.1111/j.1365-294X.2005.02553.x 15969739

[B44] Team RDC R: A Language and Environment for Statistical Computing 2009 R Foundation for Statistical Computing, Vienna, Austria

