# Supplementary material for: Patterns of molecular and phenotypic diversity in pearl millet [Pennisetum glaucum (L.) R. Br.] from West and Central Africa and their relation to geographical and environmental parameters
Source: BMC Plant Biol. 2010 Oct 6;10:216. doi: 10.1186/1471-2229-10-216 (PMC3017833; doi:10.1186/1471-2229-10-216)
Supplement: Additional file 1 — Analysis of molecular variance for the 145 pearl millet inbred genotypes of this study. Analysis of molecular variance for the 145 pearl millet inbred genotypes with respect to their country and agro-ecological zone of origin, where DF are the degrees of freedom, SSD the sum of squares deviations, σ2 the variance component, and % the percentage of variance contributed by each source of variation. [file 1471-2229-10-216-S1.PDF]

Hierarchy level	Source of variation	DF	SSD	σ^2	%
Country	Among countries	9	128.82	0.053	0.85
	Among landraces within countries	113	1438.21	4.895	79.42
	Within landraces	167	203.00	1.216	19.72
Agro-ecological zone	Among agro-ecological zones	2	30.58	0.016	0.25
	Among landraces within agro-ecological zones	120	1536.45	4.927	80.01
	Within landraces	167	203.00	1.216	19.74
