# Supplementary figures and images for: Patterns of molecular and phenotypic diversity in pearl millet [Pennisetum glaucum (L.) R. Br.] from West and Central Africa and their relation to geographical and environmental parameters
Source: BMC Plant Biol. 2010 Oct 6;10:216. doi: 10.1186/1471-2229-10-216 (PMC3017833; doi:10.1186/1471-2229-10-216)

$K = 2$

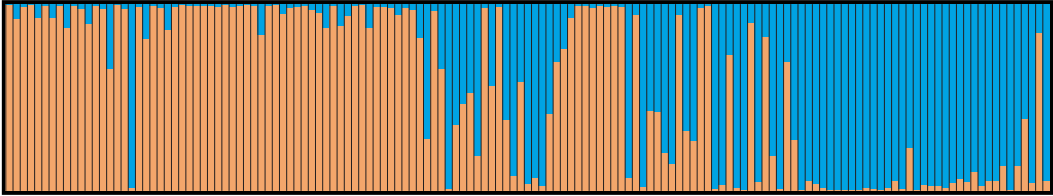

$K = 3$

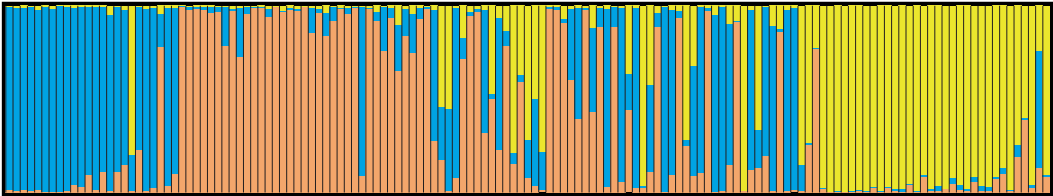

$K = 4$

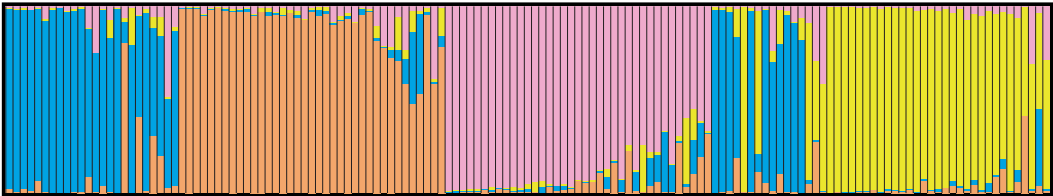

$K = 5$

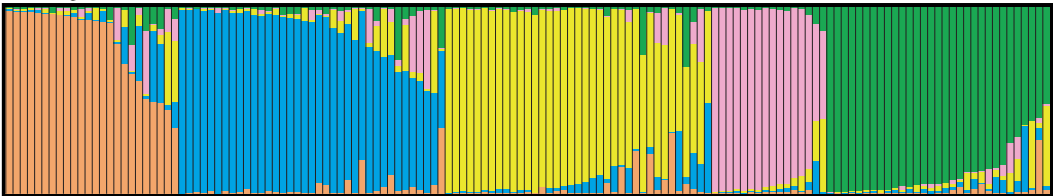

$K = 6$

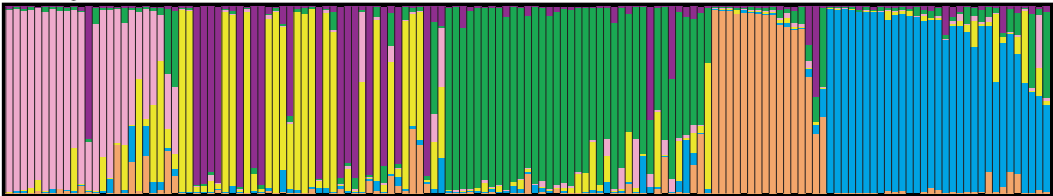

Supplement: Additional file 2 — Graphical representation of the results of STRUCTURE. Graphical representation of the results of STRUCTURE, where K is the number of sub-groups. [file 1471-2229-10-216-S2.PDF]

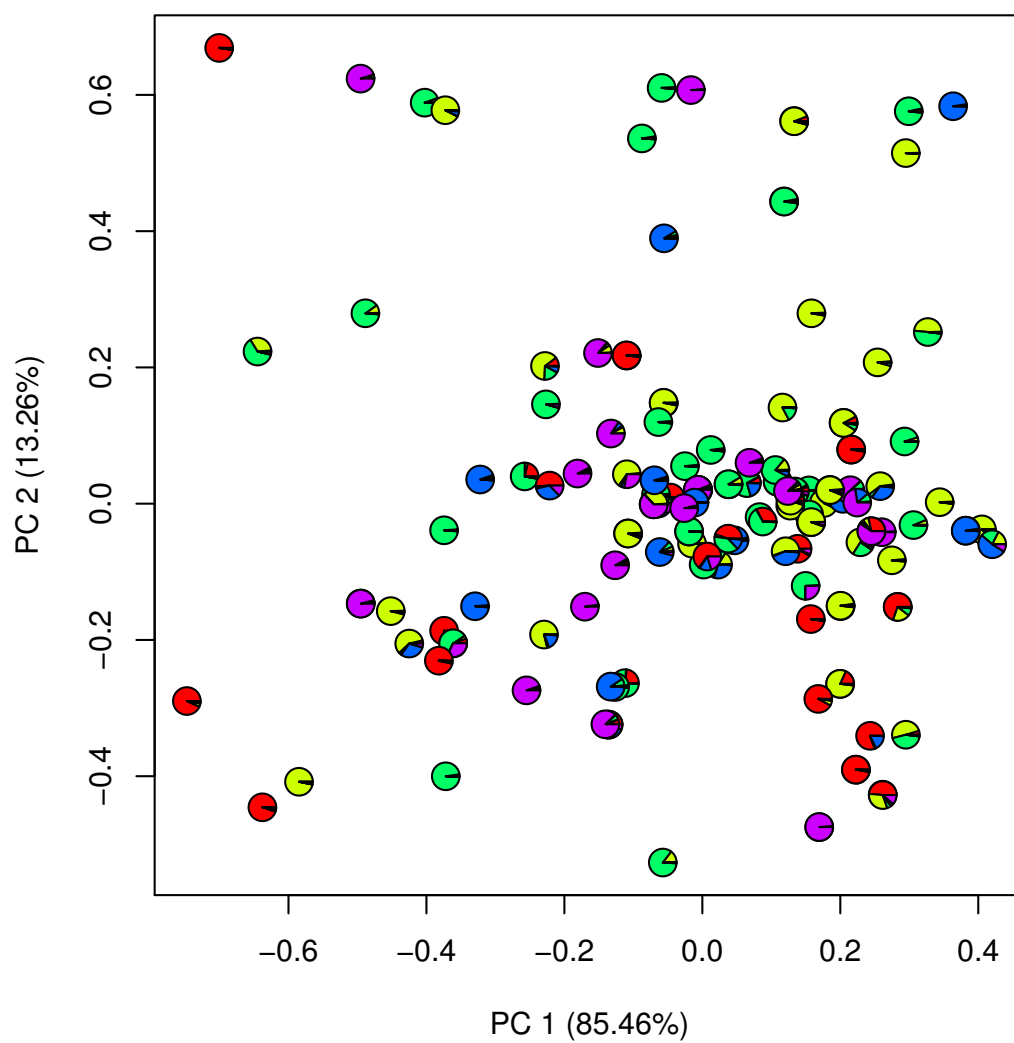

Supplement: Additional file 6 — Principal component analysis of the 145 pearl millet inbreds examined in our study based on the corresponding geographical and environmental parameters as well as the evaluated phenotypic traits. Principal component analysis of the 145 pearl millet inbreds examined in our study based on the corresponding geographical and environmental parameters as well as the evaluated phenotypic traits. PC1 and PC2 are the first and second principal component, respectively, and the values in brackets give the proportion of explained variance. The different colored segments of the pie charts give the probability that a certain individual belongs to one of the five sub-groups identified by STRUCTURE. [file 1471-2229-10-216-S6.PDF]
