# Supplementary material for: Patterns of molecular and phenotypic diversity in pearl millet [Pennisetum glaucum (L.) R. Br.] from West and Central Africa and their relation to geographical and environmental parameters
Source: BMC Plant Biol. 2010 Oct 6;10:216. doi: 10.1186/1471-2229-10-216 (PMC3017833; doi:10.1186/1471-2229-10-216)
Supplement: Additional file 4 — Simple sequence repeats markers used in this study. Simple sequence repeats markers used in this study, where LG is the linkage group, Pos. the position in cM, and Topt the optimized annealing temperature. [file 1471-2229-10-216-S4.PDF]

Marker	LG	Pos.	Primer forward ($5' - 3'$)	Primer reverse ($5' - 3'$)	Repeat motif	T_{opt}	Reference
PSMP2001	5	16	CATGAAGCCAATTAGGTCTC	ACCATCTGACTTGTTCTTATCC	(CT)(CA)	50	(36)
PSMP2008	5	44	GATCATGTTGTCATGAATCACC	ACACTACACCTACATACGCTCC	(GT)	60	(36)
PSMP2027	7	81	AGCAATCCGATAACAAGGAC	AGCTTTGGAAAAGGTGATCC	(GT)	50	(36)
PSMP2030	1	35	ACCAGAGCTTGGAAATCAGCAC	CATAATGCTTCAAATCTGCCACAC	(CA)(GA)	50	(36)
PSMP2043	7	31	TCATATTCTCCTGTCTAAAACGTC	ACAAATCGTACAAGTTCCACTC	(CA)	55	(36)
PSMP2063	7	53	GAGCACATGAAATAGGAAG	AAGGTAGTTATAGTTAGCTTGATC	(AC)(AT)	55	(36)
PSMP2071	3	14	TTGCAGTCCCACGAATTATTTG	CTATGAATTTATAATCCTGATACT	(CA)	55	(36)
PSMP2076	4	33	GGAATAGTATATTGGCAAAATGTG	ATACTACACCTGTAAGCATTGTC	(AC)	50	(36)
PSMP2080	1	-	CAGAATCCCCACATCTGCAT	TGCAACTGAGCGAAGATCAA	(AC)	50	(36)
PSMP2085	5	38	GCACATCATCTCTATAGTATGCAG	GCATCCGTCATCAGGAAATAA	(AC)	50	(36)
PSMP2087	7	54	GGAACAGACTCCATACCTGAAA	TACCTGCCTGTGCTGTTAGT	(AC)	50	(36)
PSMP2090	1	108	AGCAGCCCAGTAATACCTCAGCTC	AGCCCTAGCGCACAACACAAACTC	(CT)	55	(36)
PSMP2208	5	28	GGAAGAGCAAACTGAACAATCCC	ACTTTGCCCTGGATGATCCTC	(GT)	50	(35)
PSMP2237	2	35	TGGCCTTGGCCTTTCCACGCTT	CAATCAGTCCGTAGTCCACACCCCA	(GT)	50	(34)
PSMP2246	1	34	CGGATGCTAAATTAACCGAAGC	CCAGCTTGCTTCTGTTGCGTTC	(TG)	55	(34)
PSMP2248	6	51	TCTGTTTGTTTGGGTCAGGTCCTTC	CGAATACGTATGGAGAACTGCGCATC	(TG)	55	(34)
PSMP2249	3	-	CAGTCTCTAACAAACAAACACGGC	GACAGCAACCAACTCCAAACTCCA	(GT)	55	(34)
PSMP2267	3	115	GGAAGGCGTAGGGATCAATCTCAC	ATCCACCCGACGAAGGAAACGA	(GA)	55	(34)
PSMP2275	6	-	CCAGTGCCTGCATTCTTGGC	GCATCGAATACTTCATCTCA	(AAG)	50	MD Gale, pers. comm.
ICMP3002	6	-	CGAGCCGCCATAGTTGAC	TACACACACATTGCCACACG	(AAG)	50	(37)
