# Supplementary material for: Patterns of molecular and phenotypic diversity in pearl millet [Pennisetum glaucum (L.) R. Br.] from West and Central Africa and their relation to geographical and environmental parameters
Source: BMC Plant Biol. 2010 Oct 6;10:216. doi: 10.1186/1471-2229-10-216 (PMC3017833; doi:10.1186/1471-2229-10-216)
Supplement: Additional file 5 — Screen shot of the MegaBACE Fragment Profiler. Screen shot of the MegaBACE Fragment Profiler to illustrate the procedure of allele calling. [file 1471-2229-10-216-S5.PPT]

## Slide 1
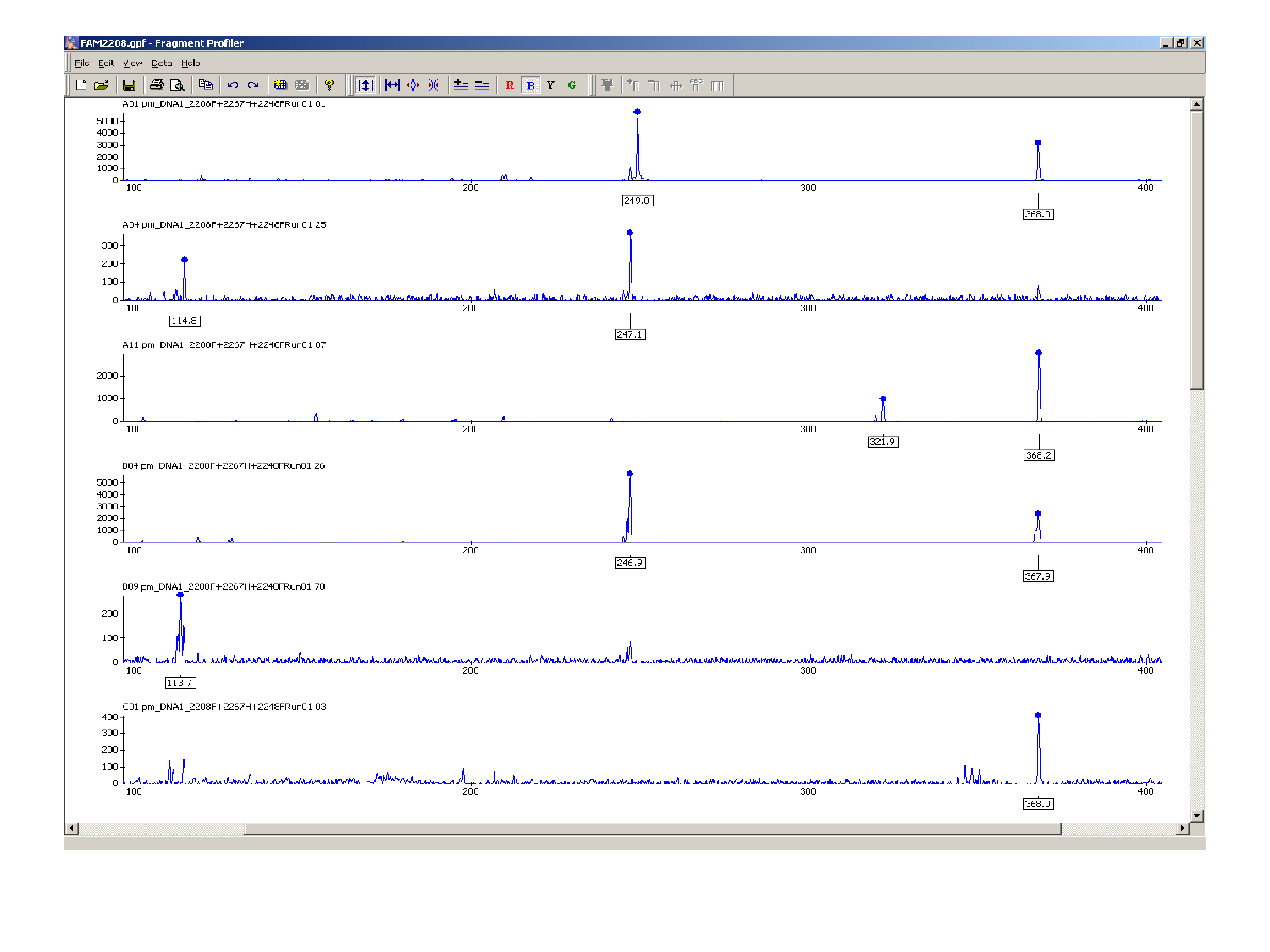

#

## Slide 2
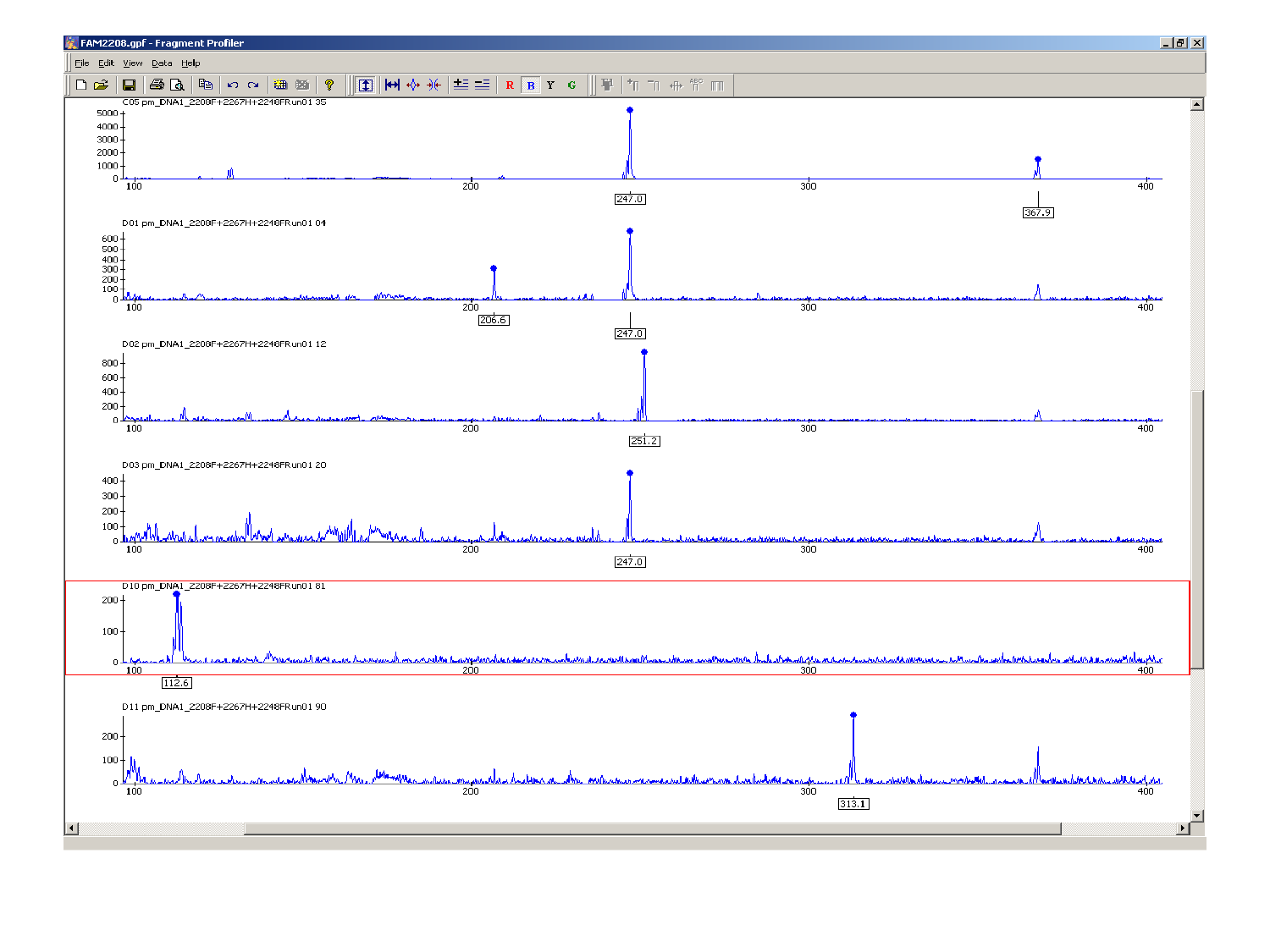

#

## Slide 3
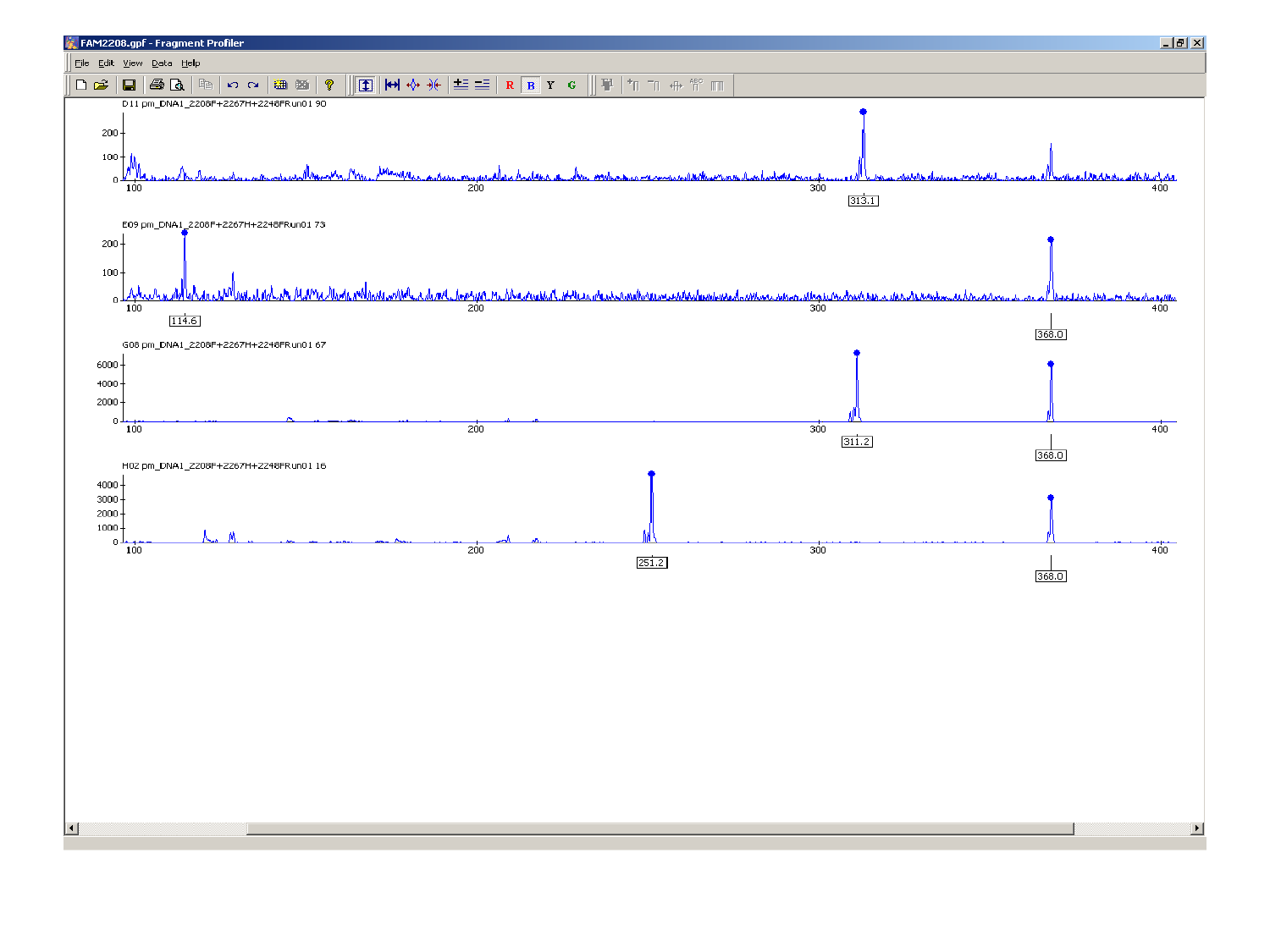

#

## Slide 4
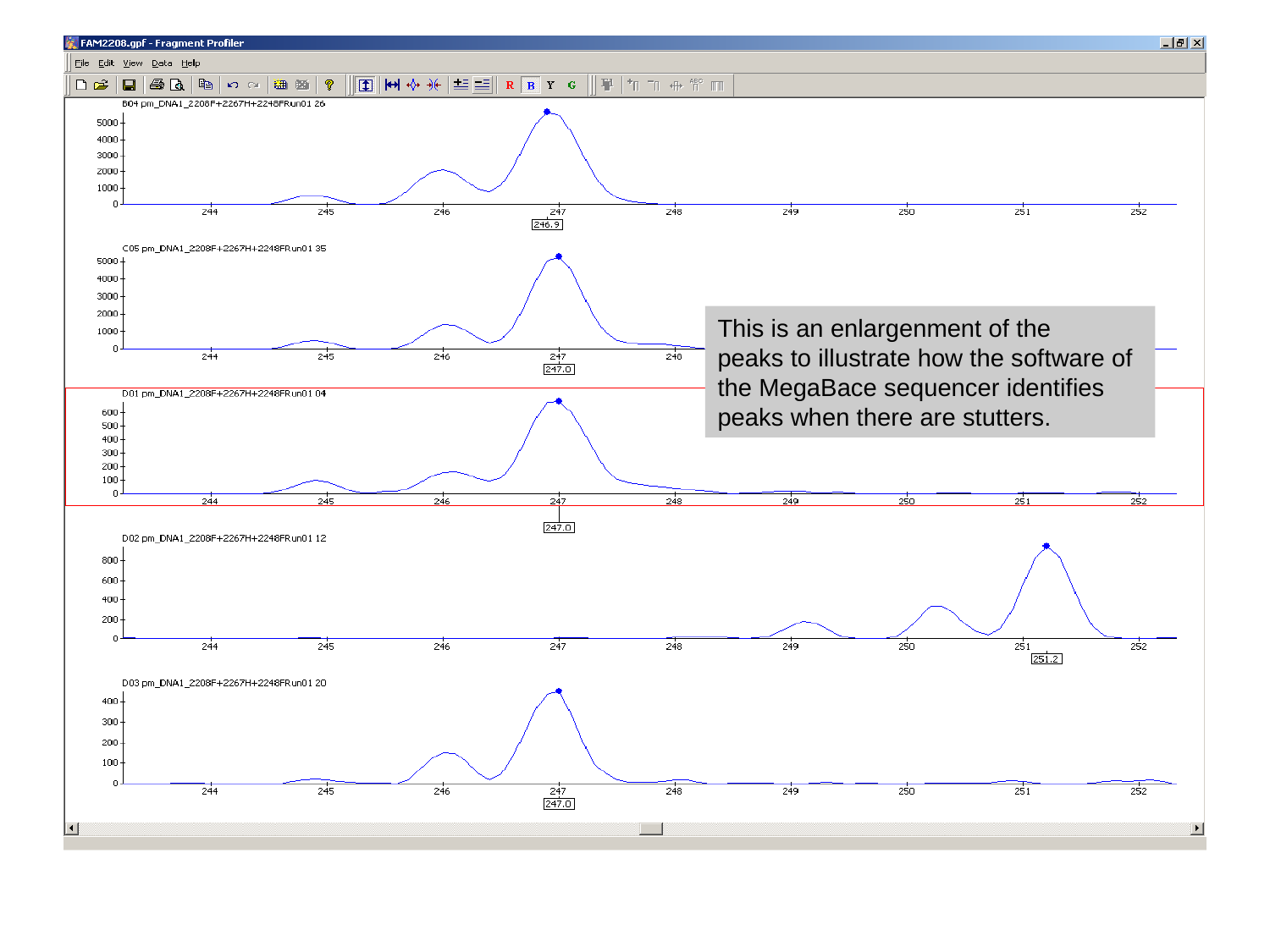

#
This is an enlargenment of the
peaks to illustrate how the software of the MegaBace sequencer identifies peaks when there are stutters.
